# Knochentransplantation oder Biomaterial? Eine Analyse von 99.863 Operationen in Orthopädie und Unfallchirurgie aus dem Jahr 2018 in Deutschland

**DOI:** 10.1007/s00113-020-00861-z

**Published:** 2020-09-04

**Authors:** Markus Rupp, Maximilian Kerschbaum, Lisa Klute, Leona Frank, Volker Alt

**Affiliations:** Klinik und Poliklinik für Unfallchirurgie, Universitätsklinikum Regensburg, Franz-Josef-Strauß-Allee 11, 93053 Regensburg, Deutschland

**Keywords:** Knochenersatzmaterial, Knochenzement, Keramischer Knochenersatz, Autograft, Allograft, Bone graft substitute, Bone cement, Ceramic bone void fillers, Autograft, Allograft

## Abstract

**Fragestellung:**

Ziel der Arbeit war es, (1) die Gesamtzahl der operativen Eingriffe mit autologer bzw. allogener Knochentransplantation sowie Biomaterialien zu analysieren. (2) Es sollten die unterschiedlichen Arten von Biomaterialien, autologen und allogenen Knochentransplantationen untersucht und (3) die zusätzliche Anwendung eines Antibiotikumzusatzes bei Biomaterialien analysiert werden.

**Methodik:**

Daten wurden vom Statistischen Bundesamt für das Jahr 2018 bezogen. Durch die Operationen- und Prozedurenschlüssel 5‑784 „Knochentransplantation und -transposition“ und 5‑785 „Implantation von alloplastischem Knochenersatz“ konnten die Prozedurhäufigkeit der Implantation von Biomaterialien, wie Kalziumphosphatkeramiken, Kalziumsulfate, Kalziumphosphatzemente und Polymethylmethacrylat, sowie autologem und allogenem Knochen zur Defektrekonstruktion an Extremitäten und Becken analysiert werden.

**Ergebnisse:**

Im Jahr 2018 wurden insgesamt 99.863 Prozeduren unter Verwendung von Autografts (54.784, 55 %). Biomaterialien (23.838, 24 %) und Allografts (21.241, 21 %) durchgeführt. Sowohl bei Autografts als auch bei Allografts kamen am häufigsten Spongiosaplastiken (77 % resp. 79 %) zum Einsatz. Bei den Biomaterialien wurden Keramiken (42 %) häufiger als Knochenzemente (37 %) benutzt (sonstige Biomaterialien 21 %). Bei 16.027 (67 %) der Biomaterialien wurde kein Antibiotikumzusatz verwendet. Antibiotikumzusatz kam v. a. bei Knochenzementen zum Einsatz (6612 Fälle, 75 %).

**Schlussfolgerung:**

Im Jahr 2018 wurden insgesamt bei einer beträchtlichen Anzahl von 99.863 Eingriffen zur Knochendefektrekonstruktion Knochentransplantate bzw. Biomaterialien eingesetzt. Bei mehr als der Hälfte der Fälle wurde autologer Knochen (55 %), ungefähr einem Viertel Biomaterialien (24 %) und etwa einem Fünftel (21 %) allogener Knochen eingesetzt. Als Biomaterial wurden Keramiken (42 %) öfter als Zemente (37 %) verwendet. Antibiotikumzusatz kam v. a. bei Zementen zum Einsatz (75 %).

## Abstract (en)

**Background:**

The aims of this study were (1) to analyze the total number of interventions with autologous or allogeneic bone transplantation as well as biomaterials, (2) to investigate the different types of biomaterials, autologous and allogeneic bone transplantations and (3) to analyze the additional use of an antibiotic additive in biomaterials.

**Methods:**

Data were obtained from the Federal Statistical Office for the year 2018. The surgery and procedure codes 5‑784 “bone transplantation and transposition” and 5‑785 “implantation of alloplastic bone substitutes” were used to analyze the procedure frequency of implantation of biomaterials, such as calcium phosphate ceramics, calcium sulphate, calcium phosphate cements and polymethyl methacrylate as well as autologous and allogeneic bone for defect reconstruction of the extremities and pelvis.

**Results:**

In 2018 a total of 99,863 procedures were performed using autografts (54,784, 55%), biomaterials (23,838, 24%) and allografts (21,241, 21%). For both autografts and allografts cancellous bone grafting was used most frequently (77% and 79%, respectively). For biomaterials, ceramics (42%) were used more frequently than bone cements (37%, other biomaterials 21%). In 16,027 cases (67%) with biomaterials no antibiotic supplement was used. Antibiotic supplementation was mainly used for bone cements (6612 cases, 75%).

**Conclusion:**

In 2018 bone transplants or biomaterials were used in a considerable number of the 99,863 bone defect reconstruction procedures. Autologous bone was used in more than half of the cases (55%), biomaterials in approximately one quarter (24%) and allogenic bone in approximately one fifth (21%). Ceramics (42%) were more often used as biomaterials than cements (37%). The addition of antibiotics was mainly used with cements (75%).

## Hintergrund und Fragestellung

Die Implantation von Knochenersatzmaterialien und die Transplantation von autologem oder allogenem Knochen zur Knochendefektrekonstruktion sind aus der modernen orthopädisch unfallchirurgischen Therapie nicht wegzudenken. Das Auffüllen von knöchernen Defekten ist bei einer Vielzahl von Indikationen notwendig. Osteoporotische Frakturen, welche durch die demografische Entwicklung der Gesellschaft immer häufiger werden, führen häufig zu v. a. metaphysären knöchernen Defekten [6]. Traumatische Defekte resultieren außerdem aus Hochrasanztraumata. Zudem bedarf es auch bei Osteotomien und bei der Behandlung von Tumoren und Infektionen des Knochens der Knochendefektrekonstruktion [17, 20]. Bis heute ist die autologe Knochentransplantation der Goldstandard hierfür. Autologer Knochen bietet alle für die knöcherne Heilung notwendigen Merkmale. Dem autologen Transplantat sind neben einer knöchernen Matrix als Leitstruktur (Osteokonduktion) Zellen (Osteogenese) und Wachstumsfaktoren (Osteoinduktion) zu eigen [8]. Nachteile der autologen Knochentransplantation sind die begrenzte Verfügbarkeit, die Entnahmemorbidität und eine verlängerte Operationszeit, die aus der Entnahme resultiert [5]. Aus diesem Grund werden als Alternativen zur autologen Knochentransplantation Spenderknochen (Allografts) und Biomaterialien angewendet. Allografts haben den Nachteil, dass sie lediglich osteokonduktiven Charakter und keine osteogene sowie allenfalls nur sehr geringe osteoinduktive Potenz besitzen [3]. Zudem lastet der Allografttransplantation die Gefahr der Übertragung viraler Infektionen wie Hepatitis oder humaner Immundefizienz-Viren an, obwohl nach Verbesserung der Testmethoden in den 1990er-Jahren keine Fälle diesbezüglich mehr berichtet wurden [18]. Ein weiterer Nachteil von Allografts und gleichfalls auch von Knochenersatzmaterialien ist die bakterielle Kontamination mit Infektion des Fremdmaterials. Für Allografts sind Kontaminationsraten bis zu 12,4 % beschrieben [13]. Der Infektionsgefahr wird neben dem Einsatz von systemisch wirksamen Antibiotika mit der zusätzlichen Anwendung lokaler Antibiotika Rechnung getragen. Außer zur Infektprophylaxe ist deren therapeutischer Nutzen bei Knocheninfektionen belegt [12, 16]. Aufgrund der Nachteile, welche der Transplantation von Auto- und Allografts anlasten, werden seitens der Wissenschaft, aber auch von der Industrie immense Anstrengungen unternommen, ein dem Goldstandard entsprechendes Knochenersatzmaterial mit osteogenem, osteokonduktivem und osteoinduktivem Potenzial zu entwickeln. Trotz dieser Bemühungen und der mittlerweile breiten Anwendung von Biomaterialien im klinischen Alltag gibt es keine publizierten Daten über die Häufigkeit der Anwendung der einzelnen Biomaterialien und Knochentransplantate in Deutschland. Daher wurden folgende Fragestellungen definiert: (1) Wie oft wurden 2018 Eingriffe mit autologer bzw. allogener Knochentransplantation sowie Biomaterialien durchgeführt? (2) Welche unterschiedlichen Arten von Biomaterialien, autologen und allogenen Knochentransplantationen wurden verwendet? (3) Wie oft wurde bei Biomaterialien ein Antibiotikumzusatz angewendet?

## Studiendesign und Untersuchungsmethoden

Da es sich bei der zu beantwortenden Fragestellung um Eingriffe handelt, welche in der Regel in stationärem Rahmen stattfinden, wurde für die Datenanalyse das Statistische Bundesamt, Wiesbaden, angefragt, um die aktuellsten Daten eines Jahres bereitzustellen. Alle in stationärem Rahmen durchgeführten Operationen und Prozeduren werden durch das Statistische Bundesamt anhand des Operationen- und Prozedurenschlüssels (OPS) erfasst, welcher bei jedem operativen Eingriff für die durchgeführten Maßnahmen zu dokumentieren ist. Der aktuellste Datensatz, welcher zum Zeitpunkt der Datenabfrage im Februar 2020 verfügbar war, war die vollständige Erfassung der Verwendung von Knochenersatzmaterialien und Knochentransplantationen für das Jahr 2018. Es wurde sich auf die Lokalisation der knöchernen Rekonstruktion an den Extremitäten und am Becken beschränkt, welche allesamt unter dem übergeordneten OPS-Code 5‑784 und 5‑785 dokumentiert wurden. Diese beinhalten jegliche Art von Knochentransplantation und -transposition (5-784), schließen aber auch zugleich den Verschluss oder die Verfüllung von iatrogen geschaffenen oder zugangsbedingten Knochendefekten mit ortsständigem Gewebe aus. Die Implantation von alloplastischem Knochenersatz (5-785) schließt alle Eingriffe zu endoprothetischem Gelenk- und Knochenersatz aus. Bei Knochenersatzmaterialien wurde anhand der OPS-Codes zwischen Knochenzementen ohne (5-785.0) und mit Antibiotikumzusatz (5-785.1) unterschieden. Bei den keramischen Knochenersatzmaterialien wurden zwischen Keramiken mit Antibiotikumzusatz (5-785.5) und solchen ohne (5-785.2 und 5‑785.3) unterschieden. Weitere Codes wie metallische Knochenersatzmaterialien (5-785.4) und sonstige alloplastische Knochenersatzmaterialien ohne (5-785.6) und mit (5-785.7) Antibiotikumzusatz wurden ebenfalls in die Analyse miteingeschlossen. Hinsichtlich der Knochentransplantationen wurden autologe Verfahren (autologe Spongiosa (5-784.0, 5‑784.c), autologer Knochenspan (5-784.1, 5‑784.2, 5‑784.d), sonstige autologe Knochentransplantationen (5-784.3, 5‑784.4, 5‑784.5, 5‑784.a)) mit allogenen Verfahren verglichen (allogene Spongiosa (5-784.7, 5‑784.9, 5‑784.e), allogener Span (5-784.8, 5‑784.f), humane demineralisierte Knochenmatrix (5-784.b) und sonstige allogene Knochentransplantation (5-784.6)) (Tab. 1).

**Table 1:** 

OPS-Code	Art des verwendeten Materials
5‑784.0, 5‑784.c	Autologe Spongiosa
5‑784.1, 5‑784.2, 5‑784.d	Autologer Knochenspan
5‑784.3, 5‑784.4, 5‑784.5, 5‑784.a	Sonstige autologe Knochentransplantation
5‑784.7, 5‑784.9, 5‑784.e	Allogene Spongiosa
5‑784.8, 5‑784.f	Allogener Span
5‑784.b	Humane demineralisierte Knochenmatrix
5‑784.6	Sonstige allogene Knochentransplantation
5‑785.0	Knochenzement ohne Antibiotikumzusatz
5‑785.1	Knochenzement mit Antibiotikumzusatz
5‑785.2, 5‑785.3	Keramischer Knochenersatz ohne Antibiotikumzusatz
5‑785.5	Keramischer Knochenersatz mit Antibiotikumzusatz
5‑785.4, 5‑785.6	Metallischer Knochenersatz. sonstige alloplastische Knochenersatzmaterialien ohne Antibiotikumzusatz
5‑785.7	Sonstige alloplastische Knochenersatzmaterialien mit Antibiotikumzusatz

*OPS* Operationen- und Prozedurenschlüssel

Die Daten wurden unter Verwendung der Statistik Software SPSS Version 26.0 (IBM, SPSS Inc. Armonk, NY, USA) analysiert und grafisch dargestellt.

## Ergebnisse

**Figure 1:**
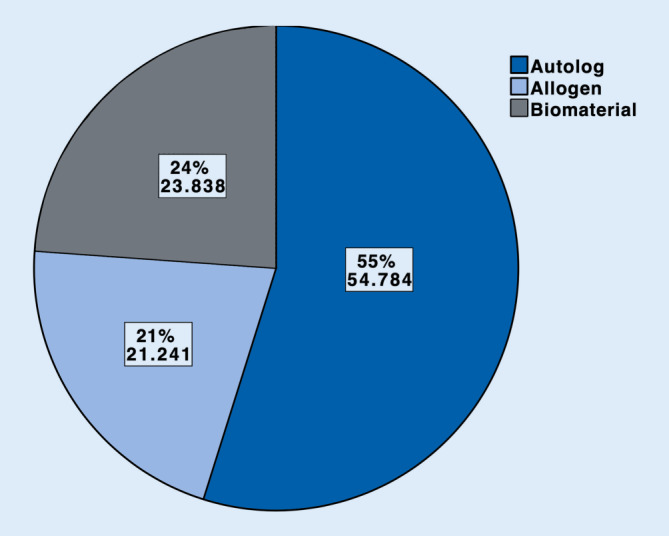

**Figure 2:**
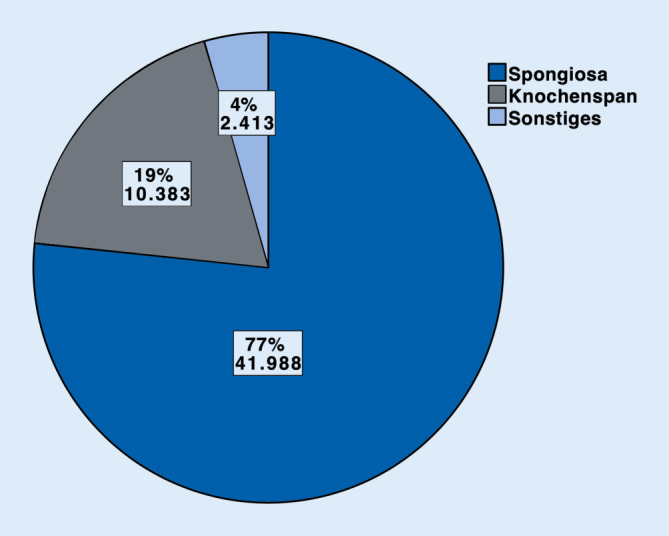

**Figure 3:**
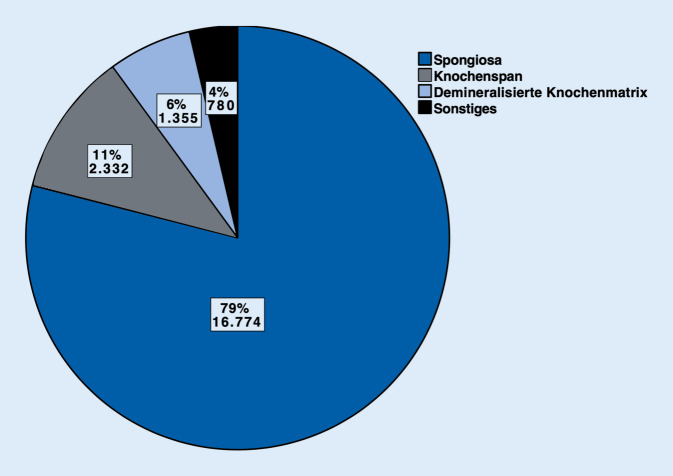

**Figure 4:**
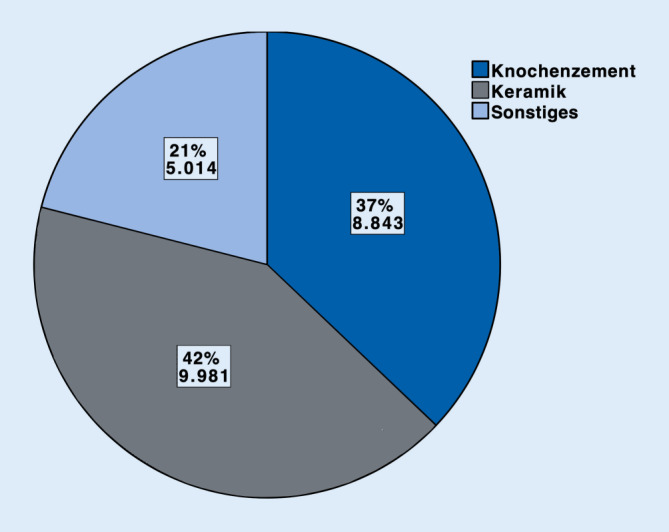

**Figure 5:**
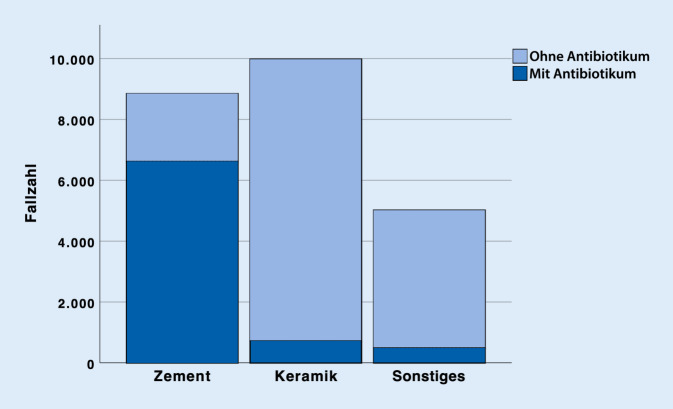

**Figure 6:**
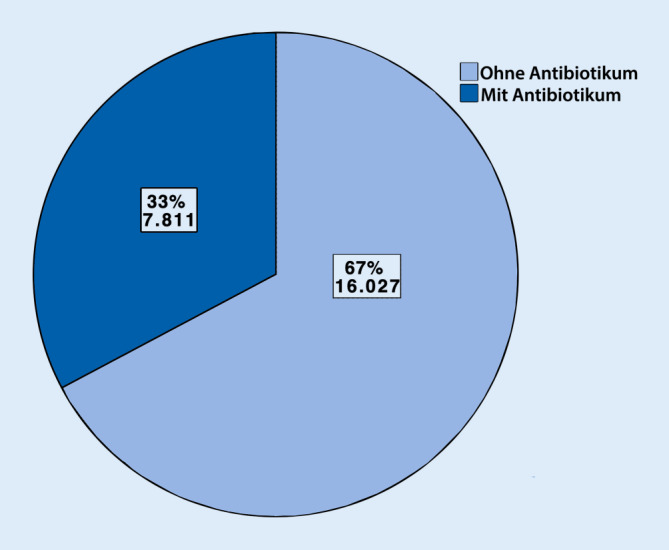

Im Jahr 2018 wurden insgesamt 99.863 operative Prozeduren verschlüsselt, die die Verwendung von autologem, allogenem Knochenersatzmaterial oder Biomaterialien an Extremitäten und Becken beinhalten. Mehr als die Hälfte (54.784, 55 %) der verwendeten Materialien zur Knochendefektrekonstruktion waren autolog. Allogener Knochen wurde hingegen am seltensten verwendet (21.241, 21 %). Biomaterialien kamen insgesamt in 23.838 Fällen (24 %) zum Einsatz (Abb. 1). Betrachtet man die einzelnen Arten der autologen Knochentransplantation, so wurde in 41.988 Fällen, sprich 77 % aller autologen Transplantationen, Spongiosa verwendet. In 10.383 Fällen (19 %) wurden Knochenspäne angewendet. In 2413 Fällen (4 %) wurden sonstige Arten der autologen Knochentransplantation codiert (Abb. 2). Bei den Allografts wurden in 79 % der Fälle (*n* = 16.774) Spongiosa, in 11 % Knochenspäne (*n* = 2332) und in 6 % der Fälle (*n* = 1355) demineralisierte Knochenmatrix verwendet. Bei 4 % wurden sonstige allogene Knochentransplantationen dokumentiert (*n* = 780; Abb. 3). Bei der Verwendung von Biomaterialien zur Knochendefektrekonstruktion wurden am meisten keramische Knochenersatzstoffe verwendet (*n* = 9981; 42 %). Zemente wurde in 8843 Fällen, sprich 37 % aller Biomaterialien, benutzt. Bei 21 % (*n* = 5014) kam es zur Anwendung von sonstigen Ersatzmaterialien (Abb. 4). Bei der Betrachtung von antibiotikahaltigen Biomaterialien wurden in der Mehrzahl der Fälle (74 %) antibiotikahaltige Knochenzemente angewendet (6612 Fälle mit Antibiotikum vs. 2231 Fälle ohne Antibiotikum). Bei keramischen Knochenersatzmaterialien ist der Anteil der Anwendung mit Antibiotikumzusatz deutlich geringer (715 Fälle; 7 %). Die überwiegende Anzahl der keramischen Biomaterialien wurde ohne Antibiotikazusatz zum Einsatz gebracht (9266 Fälle; 93 %) (Abb. 5). Insgesamt wurden bei der Anwendung von Biomaterialien antibiotikafreie Materialien in über zwei Drittel der Fälle verwendet (67 %, 16.027 Fälle). Biomaterialien mit Antibiotikumzusatz wurden hingegen in 7811 Fällen (33 %) eingesetzt (Abb. 6).

## Diskussion

Die vorliegende Studie beschreibt zum ersten Mal die Häufigkeit und Verteilung der Anwendung verschiedener Biomaterialien sowie autologer und allogener Knochentransplantationen in Deutschland. Der verwendete, vom Statistischen Bundesamt zur Verfügung gestellte, aktuelle Datensatz für das Jahr 2018 umfasst insgesamt annähernd 100.000 operative Prozeduren (99.863) an Extremitäten und Becken. Da es sich in der Regel um komplexere Eingriffe handelt, die in stationärem Rahmen durchgeführt werden und alle somatisch tätigen Kliniken verpflichtet sind, nach dem Diagnosis-Related-Groups(DRG)-System abzurechnen, ist davon auszugehen, dass die zur Verfügung gestellten Daten die orthopädische und unfallchirurgische Versorgungssituation zur knöchernen Defektrekonstruktion in Deutschland detailliert widerspiegeln.

### Die autologe Knochentransplantation ist das häufigste Verfahren zur Knochendefektrekonstruktion

Der Goldstandard der autologen Knochentransplantation macht den Großteil aller Prozeduren der knöchernen Defektrekonstruktion mit 55 % aus. Die biologischen Vorteile durch das osteogene, osteokonduktive und osteoinduktive Potenzial von autologem Knochen scheint bei der Auswahl der Therapiealternativen über die Nachteile, die eine Entnahme von autologem Knochen mit sich bringt, zu überwiegen. Insbesondere die häufig durchgeführte Entnahme von Beckenkammspänen oder -spongiosa geht mit Komplikationsraten bis zu 19,37 % einher [7]. Auch für die „Reamer-Irrigator-Aspirator“-Technik wird eine Komplikationsrate von 6 % beschrieben [7]. Erwähnte Gefahren einer viralen Infektion oder einer bakteriellen Kontamination eines Allografts könnten wiederum ursächlich sein, dass diese zusätzlich zu deren schlechteren biologischen Eigenschaften im Vergleich zu autologem Knochen weniger zum klinischen Einsatz kommen. Dass Knochenersatzmaterialien häufiger als Allografts zur Anwendung kommen, kann an der durch die Vielzahl der auf dem Markt verfügbaren Produkte, Materialkombinationen und den von der Industrie offensiv beworbenen Vorteilen synthetischer Knochenersatzmaterialien liegen [9]. Die verhältnismäßig häufigere Anwendung von Biomaterialien gegenüber Allografts entspricht auch den Erwartungen für den weltweiten Markt für Knochentransplantate und Knochenersatzmaterialien bis zum Jahr 2025, welcher dann auf 3,912 Mrd. US-Dollar geschätzt wird [2]. Die im stationären Rahmen nach DRG-System vergüteten zusätzlichen Prozeduren zur Knochendefektrekonstruktion führen je nach ursprünglichem Eingriff zu unterschiedlichen Veränderungen in der Vergütung der operativen Leistung. Etwaige monetäre Ursachen für die Anwendung der einzelnen Verfahrensarten lassen sich daher nicht sicher ableiten.

### Spongiosaplastiken sind das „Arbeitspferd“ der autologen und allogenen Knochentransplantation

Sowohl bei autologer als auch bei allogener Knochentransplantation sind Spongiosaplastiken die am meisten codierte Art der Defektrekonstruktion (41.988 Fälle; 77 % resp. 16.774 Fälle; 79 %) (Abb. 2 und 3) Die bei osteoporotischen Frakturen häufiger resultierenden metaphysären Defekte bedürfen einer Augmentation des nicht mehr oder nur noch wenig vorhandenen spongiösen Knochens, während diaphysäre Defekte, wie sie bei Hochrasanztraumata auftreten können, eher einer kortikalen Defektrekonstruktion bedürfen [4]. Entscheidend ist dabei die Form der Defekte und deren Häufigkeit. Nichtsegmentale Defekte lassen sich gut mit Spongiosa auffüllen, solange noch genügend Kortikalis vorhanden ist, die ein Containment des Knochentransplantats bewerkstelligt. Je nach anatomischer Position wird bei segmentalen Defekten zwischen 2,5 und 6 cm mit guten Therapieaussichten für eine Knochentransplantation ausgegangen [11, 14]. Hier kommen kortikale Transplantate eher zum Einsatz, da diese eine zusätzliche Stabilität liefern können. Insgesamt sind kleinere Knochendefekte mit der Möglichkeit spongiöser Augmentation häufiger, was die überwiegende Anzahl autologer und allogener Spongiosaimplantationen erklären kann.

### Bei einem Drittel der Biomaterialien wird ein Antibiotikumzusatz angewendet

Bei den Knochenersatzmaterialien zeigt sich, dass bei den zur Analyse differenzierten Gruppen v. a. Keramiken (42 %) und Knochenzemente (37 %) zum Einsatz kamen. Bei einem Drittel wurden Materialien mit Antibiotikumzusatz gewählt (Abb. 6). Insbesondere bei Knochenzementen ist der Anteil antibiotikumhaltiger Materialien höher (74 %) als bei Keramiken (7 %). Aus der endoprothetischen Versorgung ist die Anwendung von antibiotikumhaltigem Polymethylmethacrylat(PMMA)-Knochenzement nicht wegzudenken und hat wesentlich zu Prophylaxe und Therapie periprothetischer Infektionen beigetragen [15, 19]. Das in der klinischen Routine vertraute Implantieren von PMMA kann als Grund für die recht hohe Anzahl an Knochenzementen angesehen werden. Aber auch die biomechanischen Vorteile von PMMA und die gute Biokompatibilität trotz gleichzeitigem Fehlen jeglicher Osteokonduktion, Osteoinduktion und osteogener Potenz können als Ursache für die beinahe gleichhäufige Anwendungszahl im Vergleich zu keramischen Knochenersatz angesehen werden. Zudem ist mittlerweile die Augmentation von osteoporotischem Knochen im Rahmen der Frakturversorgung gut etabliert. Hierbei kommt regelhaft PMMA zum Einsatz [10].

Bei der Analyse von Knochenersatzmaterialien mit zusätzlichem Antibiotikumzusatz können die Marktverfügbarkeit und zugleich die Erfahrung, die Chirurgen mit PMMA im Rahmen endoprothetischer Eingriffe gesammelt haben, ursächlich dafür sein, dass ein relativer hoher Anteil an Knochenzementen mit Antibiotikumzusatz verwendet wurde. Als zugelassene keramische Knochenersatzmaterialien mit Antibiotikumzusatz ist aktuell lediglich Cerament G oder Cerament V (Fa. Bonesupport, Lund, Schweden) verfügbar, was den geringeren Antibiotikumanteil bei keramischen Knochenersatzmaterialien erklären kann. Bei sonstigen Ersatzverfahren, zu denen auch die Defektrekonstruktion mit metallischen Implantaten, wie beispielsweise Tantalimplantaten am Becken oder Diaphysenimplantaten bei langen Röhrenknochen, zählt, ist der Anteil von Antibiotikumzusatz gering. Gerade bei aufwendigen Rekonstruktionen wäre jedoch auch bei Verwendung von metallischen Implantaten eine lokale antibiotische Infektprophylaxe wünschenswert. In der Implantatentwicklung wurde bereits die Notwendigkeit von antimikrobiellen Implantatbeschichtungen zur Implantatinfektreduktion erkannt. Verschiedene antimikrobielle Implantatbeschichtungen wie ein gentamicinbeschichteter Tibiamarknagel (Expert Tibianagel PROtect; Fa. Synthes, Zuchwil, Schweiz) oder silberbeschichtete Tumorprothesen (MUTARS®; Fa. Implantcast, Buxtehude) sind in der klinischen Praxis verfügbar [1]. Allerdings spielen antibiotikahaltige Beschichtungen bei den Defektrekonstruktionen offensichtlich noch keine Rolle.

### Limitationen

Die Studie hat mehrere Limitationen. Die Verwendung von Daten, die durch das Statistische Bundesamt zur Verfügung gestellt wurden, birgt die Gefahr, dass Codierfehler, die bei der Eingabe der OPS-Codes gemacht werden, die vorgestellten Ergebnisse verzerren. Die professionelle Prüfung durch in den meisten Krankenhäusern gut ausgebildete DRG-Beauftragte spricht jedoch dafür, dass ein solcher Fehler nicht allzu groß sein sollte. Die Unterscheidung der OPS-Codes lässt eine Differenzierung zwischen den dargestellten Gruppen (Tab. 1) zu. Eine detailliertere Aufschlüsselung der in einer Fülle von verschiedenen Zusammensetzungen vorkommenden Biomaterialien wäre wünschenswert. Die einfach gehaltene Aufschlüsselung der OPS-Codes mit der Unterteilung in Zemente, Keramiken, metallische und sonstige alloplastische Materialien verringert zwar die Fehlerwahrscheinlichkeit bei der Codierung. Rückschlüsse auf bestimmte Biomaterialtrends außer den genannten Gruppen lassen sich jedoch nicht zuverlässig wiedergeben. Bei der Codierung von Knochenzement kann es sein, dass sowohl die Anwendungen von PMMA als auch von Kalziumphosphatzementen (CPC) in der gleichen Gruppe codiert wurden. Dafür spricht, dass PMMA mittlerweile nur mit Antibiotikumzusatz in Deutschland erhältlich ist und immerhin 26 % der Zementapplikationen ohne Antibiotikumzusatz codiert wurden. Auch die kombinierte Anwendung von Biomaterial, Autograft und Allograft kann durch den bereitgestellten Datensatz nicht analysiert werden. Auch wenn die aktuellen Daten des Jahres 2018 einen guten Überblick über die Materialverwendung zur Knochendefektrekonstruktion liefern können, kann daraus kein Rückschluss auf zukünftige Trends in der Applikation von Biomaterialen und Knochentransplantaten in der klinischen Praxis gegeben werden. Dieser ist abhängig von zahlreichen Faktoren wie z. B. Erfolgen in der Entwicklung neuer dem Goldstandard entsprechenden oder nahezu ähnlichen Biomaterialien, der Preisentwicklung der Biomaterialien und Allografts, deren Verfügbarkeit und nicht zuletzt deren Vergütung im DRG-System.

## Fazit für die Praxis

Die autologe Knochentransplantation ist die häufigste durchgeführte Methode zur knöchernen Defektrekonstruktion. Biomaterialen werden jedoch vor Allografts am zweithäufigsten zum Knochenersatz angewandt. Bei einem Drittel der Biomaterialien wird die lokale Antibiotikagabe favorisiert, wobei diese zumeist bei der Anwendung von Knochenzementen zum Einsatz kommt und weniger bei keramischem Knochenersatz. Weitere Verbesserungen in der Entwicklung von Knochenersatzmaterialien mit osteogenem, osteokonduktivem, osteoinduktivem und zugleich antimikrobiellem Potenzial lassen in Zukunft einen weiteren Anstieg der Anwendung von Biomaterialien im Vergleich zu Knochentransplantationen erwarten.

## References

[1] Alt V (2017). Antimicrobial coated implants in trauma and orthopaedics—a clinical review and risk-benefit analysis. Injury.

[2] Allied Market Research (2020) Bone grafts and substitutes market overview. https://www.alliedmarketresearch.com/bone-graft-substitutes-market. Zugegriffen: 23. Apr. 2020

[3] Baldwin P, Li DJ, Auston DA (2019). Autograft, allograft, and bone graft substitutes: clinical evidence and indications for use in the setting of orthopaedic trauma surgery. J Orthop Trauma.

[4] Blokhuis TJ (2017). Management of traumatic bone defects: metaphyseal versus diaphyseal defects. Injury.

[5] Calori G, Colombo M, Mazza E (2014). Incidence of donor site morbidity following harvesting from iliac crest or RIA graft. Injury.

[6] Cheung WH, Miclau T, Chow SK-H (2016). Fracture healing in osteoporotic bone. Injury.

[7] Dimitriou R, Mataliotakis GI, Angoules AG (2011). Complications following autologous bone graft harvesting from the iliac crest and using the RIA: a systematic review. Injury.

[8] Fillingham Y, Jacobs J (2016). Bone grafts and their substitutes. Bone Joint J.

[9] Heinemann S, Gelinsky M, Worch H (2011). Resorbierbare Knochenersatzmaterialien. Orthopade.

[10] Kammerlander C, Neuerburg C, Verlaan J-J (2016). The use of augmentation techniques in osteoporotic fracture fixation. Injury.

[11] Keating J, Simpson A, Robinson C (2005). The management of fractures with bone loss. J Bone Joint Surg Br.

[12] Lalidou F, Kolios G, Drosos G (2014). Bone infections and bone graft substitutes for local antibiotic therapy. Surg Technol Int.

[13] Liu J, Chao L, Su L (2002). Experience with a bone bank operation and allograft bone infection in recipients at a medical centre in southern Taiwan. J Hosp Infect.

[14] Nauth A, Schemitsch E, Norris B (2018). Critical-size bone defects: is there a consensus for diagnosis and treatment?. J Orthop Trauma.

[15] Pellegrini AV, Suardi V (2020). Antibiotics and cement: what I need to know?. Hip Int.

[16] Rupp M, Popp D, Alt V (2020). Prevention of infection in open fractures: where are the pendulums now?. Injury.

[17] Schieker M, Mutschler W (2006). Die Überbrückung von posttraumatischen Knochendefekten. Unfallchirurg.

[18] Tomford WW (2000). Bone allografts: past, present and future. Cell Tissue Bank.

[19] van Vugt T, Arts C, Geurts J (2019). Antibiotic-loaded polymethylmethacrylate beads and spacers in treatment of orthopaedic infections and the role of biofilm formation. Front Microbiol.

[20] Winkler T, Sass F, Duda G (2018). A review of biomaterials in bone defect healing, remaining shortcomings and future opportunities for bone tissue engineering: the unsolved challenge. Bone Joint Res.

