# Erratum zu: Knochentransplantation oder Biomaterial? Eine Analyse von 99.863 Operationen in Orthopädie und Unfallchirurgie aus dem Jahr 2018 in Deutschland

**DOI:** 10.1007/s00113-021-01028-0

**Published:** 2021-07-07

**Authors:** Markus Rupp, Maximilian Kerschbaum, Lisa Klute, Leona Frank, Volker Alt

**Affiliations:** Klinik und Poliklinik für Unfallchirurgie, Universitätsklinikum Regensburg, Franz-Josef-Strauß-Allee 11, 93053 Regensburg, Deutschland

**Erratum zu:**

**Unfallchirurg 2021** 124:146–152

10.1007/s00113-020-00861-z

Der Artikel „Knochentransplantation oder Biomaterial? Eine Analyse von 99.863 Operationen in Orthopädie und Unfallchirurgie aus dem Jahr 2018 in Deutschland“ von Markus Rupp, Maximilian Kerschbaum, Lisa Klute, Leona Frank und Volker Alt wurde ursprünglich Online First ohne „Open Access“ auf der Internetplattform des Verlags publiziert. Nach der Veröffentlichung in Bd. 124, Heft 2, S. 146–152 hatten sich die Autoren für eine „Open-Access“-Veröffentlichung entschieden. Das Urheberrecht des Artikels wurde deshalb in © The Author(s) 2020 geändert.

